# BioMag 1: A magnetic approach for efficient enzyme and microorganism reuse in biochemical processes for energy and food industries

**DOI:** 10.1371/journal.pone.0338444

**Published:** 2025-12-22

**Authors:** Daniela Sánchez-Orozco, Jerry Landívar, Sócrates Palacios-Ponce, Anna Ilina, Wilson Sánchez-Macías, Livingston D. Castro

**Affiliations:** 1 Facultad de Ingeniería Mecánica y Ciencias de la Producción, Escuela Superior Politécnica del Litoral, ESPOL, Campus Gustavo Galindo, km 30.5 de la vía Perimetral, Guayaquil, 090902, Ecuador; 2 Nanobioscience Group, Chemical Science School of the Autonomous University of Coahuila, Coahuila, México; 3 Faculty of Education, UNEMI: Universidad Estatal de Milagro, Milagro, Guayas, Ecuador; Adani University, INDIA

## Abstract

The production of biofuels and many food products involves biochemical processes such as hydrolysis and fermentation, which are expensive processes because of the need for new enzymes and microorganisms for each new cycle of production. Furthermore, the complexity of laboratory equipment for these tests and their cost limits the development of technological solutions for the industry. To improve these production processes, the use of open-source technologies presents a promising alternative for the development of innovative tools. In this work, we developed an automated system that enhances the efficiency of hydrolysis and fermentation assays by enabling the reuse of enzymes and microorganisms, immobilized with magnetic nanoparticles. The conceptual design of the system consisted of a reservoir with inputs and outputs to control the bioengineering process. To design the electromagnet array, it was necessary to use a mathematical model of the magnetic flux behavior of the electromagnets. With this model, it was possible to determine the magnetic density and dimensioning for the detailed design. The system required the design of controllers for the temperature, pH, and revolutions to operate simultaneously. The prototype of the mechatronic system included a user interface to control and monitor these variables in real time and to record the data during the experiment. It was possible to design and build a prototype that can use the magnetic field to pull down ferromagnetic particles within a fluid. The open-source technologies helped to build this prototype at an affordable price and opened the possibilities for further improvements. Ultimately, reducing costs and overcoming technological limitations in biofuel and food production processes.

## Introduction

Hydrolysis and fermentation are both biochemical processes that constitute crucial stages in multiple production lines in the food industry [1]. Fermentation is a metabolic process that transforms carbohydrates into alcohol or fatty acids through the action of biological systems (microorganisms such as bacteria or yeast). Almost a third of the human diet is based on products obtained through fermentation. This includes more than 5000 food products, such as dairy, cereals, jellies, vinegar, alcoholic beverages, etc. [2]. Fermentation is also needed in the production of biofuels, an industry in constant growth since it encourages the generation of renewable energy sources derived from biomass or food waste [3]. Usually, the carbohydrates involved are very complex molecules. To process these, hydrolysis is performed as a pretreatment to break them down into simpler ones. Specifically, enzymatic hydrolysis is a chemical reaction carried out by enzymes that transform complex molecular structures into monomers, facilitating the action of fermenting microorganisms [4].

In the case of the fermentation process, each cycle requires the preparation of an inoculum, which consists of the initial group of microorganisms that will reproduce and consume the carbohydrates. The time spent on the laboratory preparation process represents a large cost on the production line. In the biofuel industry, the inoculum preparation takes around 48 hours per batch. Many companies are investing in research to reduce this time to around 36 hours, representing a significant economic saving [5]. Similarly, using enzymes during the hydrolysis process implies a high cost because these enzymes are discarded in each batch. The cost of enzymes for industrial use is approximately $1000 per kilogram [6]. Depending on the kind of product, the enzymes can represent between 10% and 49% of the total cost of the process. This high rate and the need to invest in it during each iteration is a great barrier to the profitability of products involving this process, such as bioethanol and other food products [7].

To ensure the quality of the final product, the action of enzymes and fermenting microorganisms must be stopped. The most common strategies for this purpose are applying heat, changing pH, and adding chemical substances. However, this causes enzyme denaturation and the contamination or death of fermenting microorganisms [8]. Extraction processes to reuse enzymes or biological systems are too expensive, representing values from 15% to 70% of the production [9].

A new strategy for recovering enzymes and microorganisms is being developed in response to this problem. This consists of an immobilization process in which enzymes and microorganisms are linked to magnetic nanoparticles. This product will be called an “immobilized system”. This action facilitates the reuse of these elements by separating them from the liquid substance by applying a magnetic field that attracts them. The size of these nanoparticles grants them superparamagnetic behavior, which eliminates hysteresis effects. Therefore, enzymes and microorganisms can be immediately released once the external magnetic field is removed. This way, they can be separated from a previous batch and inserted into a new one to restart the hydrolysis or fermentation process [10]. Although this strategy is still in the research stage, its industrial implementation is estimated to generate savings of up to 60% in the biochemical processes mentioned [11].

Trials, research, and experiments are required to validate the efficiency of this methodology and optimize it before scaling it to an industrial level. However, progress has been delayed due to the lack of laboratory equipment for controlled tests. Currently, fermentation and hydrolysis tests are performed using bioreactors. These laboratory devices integrate control and monitoring systems for temperature, agitation, pH, and other variables relevant to the analysis of fermentation and hydrolysis tests. However, current commercial bioreactors do not allow the incorporation of a magnetic field generation system to retain magnetic nanoparticles [12]. There are devices capable of retaining magnetic particles present in liquid substances. These magnetic traps contain magnets that retain the ferrous components in the liquid flowing through them. However, this technology is not viable for the studied application. Since it relies on permanent magnets, the action of the magnetic field cannot be stopped. Once the immobilized systems are separated, they cannot be released automatically. Manual removal from the magnets would be necessary, compromising the process’s sterility. This contamination would jeopardize the reliability of the results obtained [13].

Consequently, all existing information about this process has been obtained through manual experiments performed with small glass containers or test tubes. In these, the investigator needs to approach and separate a magnet from the surface of the container. The measurement and control of variables like pH, temperature, agitation, etc., have also been done manually. This implies a slow process and a high susceptibility to human and aleatory errors. Hence, the tests present a low repetitiveness, an important experimentation characteristic [14].

The lack of specialized laboratory equipment is a very common barrier in scientific development. Due to this, a large part of the scientific community promotes the development of Open-Source technologies. These aim to facilitate knowledge transfer, allowing other laboratories and research teams to replicate and customize the provided hardware or software. Additionally, access to specialized systems facilitates and accelerates the adoption of new methodologies or techniques by the scientific community. This trend is especially beneficial for laboratories or centers with limited resources, as open hardware technology costs only between 1% and 10% of an equivalent commercial system with a private license. Therefore, countries with fewer resources should promote these types of developments [15].

This *state-of-the-art* identifies the lack of a laboratory device capable of performing controlled tests to study immobilized systems in fermentation and hydrolysis processes. The development of an open hardware alternative would contribute to the faster examination and perfection of this new technique, accelerating the laboratory tests phase to define the process’ effectiveness and industrial viability.

Therefore, this paper will present the methodology and design of the *BioMag 1*, a bioreactor prototype capable of automating the action of the magnetic field on immobilized systems, enabling their reuse. Simultaneously, the device will control the most relevant variables for these experiments: temperature, pH, and agitation. Additionally, it will allow for real-time monitoring via a graphical interface. This will show the current values of the variables mentioned. These variables will enable researchers to analyze the biochemical process throughout the entire execution cycle. The following sections will explain the decision criteria for component selection, the calculations developed, and the controllers designed for each variable. The Results section will exhibit the performance of each subsystem as part of the multi-variable control integration.

**Fig 1:**
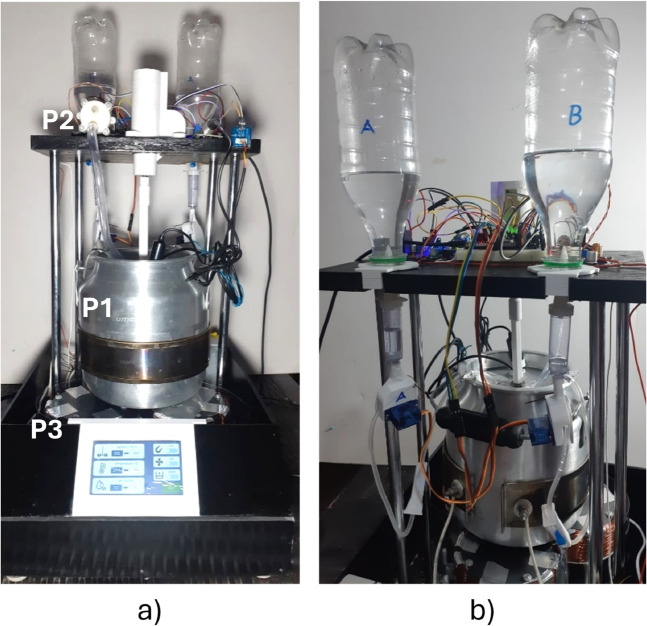
BioMag 1 prototype. A) Front view. B) Back view.

To summarize, the main contributions of this article to the scientific community are:

- A mathematical model to estimate the behavior of the magnetic flux density generated by an electromagnet array. It includes an open-source code to plot the magnetic field behavior for different design parameters.
- A methodology for the design of a multitasking system capable of controlling temperature, pH, and stirring speed. All decisions made are explained in the Materials and Methods section. The reader can adapt some initial parameters to align with their particular conditions.
- An open-source repository including plans and diagrams to build the *BioMag 1* prototype (Fig 1), and codes to solve the mathematical model and control the multitasking system.

## Materials and methods

The prototype consists of a suitable container for conducting hydrolysis and fermentation experiments. It will feature several subsystems: magnetic field control, temperature control, pH control, agitation control, and a user interface. A microcontroller will integrate all these subsystems to work simultaneously. The following sections provide detailed descriptions of each of these modules. It is important to notice that the design process followed for this project had a recurrent nature. It means that, despite presenting the information sequentially in this paper, during the development of the prototype, all subsystems were designed, tested, edited, and corrected simultaneously. This was an important part of the methodology to guarantee the correct interaction and integration of the subsystems in the multitasking device.

To resemble the product of a fermentation or hydrolysis process, carboxymethyl cellulose was added to the water, obtaining the *working substance* used for all the tests performed in this project. This substance had a high viscosity and density, similar to most of the highest possible scenarios for these biochemical processes.

**Fig 2:**
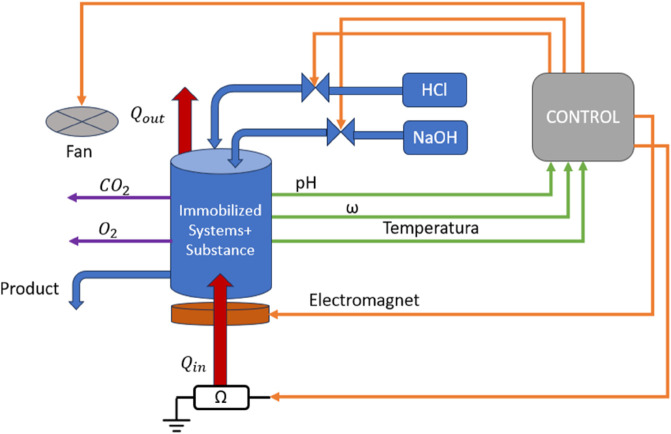
Functionality diagram. It includes all the sensors, actuators, and how the information and signals flow.

Fig 2 shows a sketch of the system’s functional design, representing all the inputs and outputs.

### Main components

The first component of the system is the container (Fig 1 - P1) where the biochemical processes must occur. In order to allow experiments with an adequate volume of the system, a 3-liter container was chosen. It has a cylindrical geometry, with a diameter of 16 cm and a height of 20 cm. The material of this container must be corrosion-resistant and autoclavable to maintain sterility during experiments. Additionally, it must be non-magnetic to avoid blocking or interfering with the magnetic field’s action on the immobilized systems. Consequently, the ideal material for this application is 304-grade austenitic stainless steel. However, due to low availability and high costs, the low-resolution prototype presented in this paper was built using an aluminum container. Despite its shorter lifespan, it meets the experimental requirements, as it is corrosion-resistant, sterilizable, and has a magnetic permeability almost equal to the air (non-magnetic).

The system’s main power supply was a TPS-1230 model from EVL, purchased locally. It is connected to the power grid (120 V - 60 Hz) to provide a variable voltage output from 5V to 15V, with a maximum current of 30A.

A discharge system was also implemented. It consisted of a silicone hose connected to a pump (Fig 1 - P2) that could drain the working substance out of the container. All the parts in contact with the liquid can be sterilized. When the electromagnetic system (explained below) is on, the immobilized biosystems will be retained while the pump extracts the liquid. Then, the container will be filled again with the working substance. This will allow the researcher to study the reuse of enzymes or microorganisms in the hydrolysis or fermentation processes. This discharge action was also automatized. A button to activate the pump is unlocked once the electromagnetic field is on. An ultrasonic sensor measures the liquid level and sends the signal to turn off the pump again.

### Magnetic field control

This subsystem generates the magnetic field that attracts the immobilized systems. It will retain them during the liquid discharge and release them when the new batch of substance arrives for the next cycle of hydrolysis or fermentation. The goal was to achieve a maximum magnetic flux density of 0.2 T inside the container at a liquid height of 10 cm. This value was selected as it is commonly used in experiments found in the literature review [14,16]. To achieve this performance, several alternatives were evaluated. The idea of using permanent magnets was discarded because they would require mechanical systems to move them away to stop the magnetic field’s action. This would unnecessarily increase the dimensions and complexity of the system. Moreover, permanent magnets limit the flexibility and scalability of the project due to their fixed magnetic strength. As a result, electromagnets were chosen.

Remembering that the magnetic field must be generated in a container with a diameter of 16 cm, it was determined that using a single electromagnet of such dimensions would be too heavy and difficult to handle. Additionally, since magnetization is not uniform, the magnetic field would not be directed correctly. For this reason, it was decided to create an array of electromagnets that collectively contribute to the total magnetic flux. According to the container dimensions, three electromagnets were arranged in a triangular configuration. This arrangement will be placed underneath the main container (Fig 1 - P3).

The following sections will describe the different stages for selecting materials and designing this subsystem.

#### Magnetic field of a coil.

The calculation of the magnetic field generated by a coil was based on (1), (2), and (3). These equations express the magnetic flux density generated on an *(x,y,z)* point, by a single loop coil with radius *a* when a current *I* flows through it.

$$B_{x}=\frac{C_{x}z}{2\alpha^{2}\beta \rho^{2}}[(a^{2}+r^{2})E(q^{2})-\alpha^{2}K(q^{2})]$$ (1)

$$B_{y}=\frac{y}{x}B_{x}$$ (2)

$$B_{z}=\frac{C_{x}z}{2\alpha^{2}\beta }[(a^{2}-r^{2})E(q^{2})+\alpha^{2}K(q^{2})]$$ (3)

The variables used in these Eqs (1), (2), and (3) can be represented in terms of *(x,y,z)* as shown below in equations from (4) to (8):

$$\rho^{2}=x^{2}+y^{2}$$ (4)

$$r^{2}=x^{2}+y^{2}+z^{2}$$ (5)

$$\alpha^{2}=a^{2}+r^{2}-2a\rho$$ (6)

$$\beta^{2}=a^{2}+r^{2}+2a\rho$$ (7)

$$q^{2}=1-\frac{\alpha^{2}}{\beta^{2}}$$ (8)

$$C=\mu_{0}\frac{I}{\pi }$$ (9)

Eqs (10) and (11) are complete elliptic integrals of the first and second kind, respectively [17].

$$K(k)=\int_{0}^{\frac{\pi }{2}}\frac{d\theta }{\sqrt{1-k^{2}\sin^{2}\theta }}$$ (10)

$$E(k)=\int_{0}^{\frac{\pi }{2}}\frac{1}{\sqrt{1-k^{2}\sin^{2}\theta }d\theta }$$ (11)

These mathematical expressions are computed using numerical methods. Note that the equations shown consider a single loop, so a multiplicative factor *N* must be added according to the number of turns in the coil.

#### Ferromagnetic core.

After performing several iterations with different parameters to dimension the electromagnets, it was determined that a ferromagnetic core was necessary. A coil core helps concentrate and enhance the magnetic flux density. The core material needs to have a high iron percentage. Iron is one of the materials with the highest magnetic permeability. Pure iron was discarded due to its fragility and difficulty to manufacture. Instead, the ASTM A36 steel was selected. As a low-carbon steel, it exhibits good magnetic properties. Its relative magnetic permeability changes depending on the provider and previous treatments (tempering, annealing, normalizing, etc.). However, this project assumed a relative permeability of 200 units. This value was estimated based on the literature review, considering the material’s carbon percentage and the approximate grain size [18,19]. This material presented the advantage of being locally available and having a relatively low cost, particularly metal sheets.

When the core experiences the coil’s magnetic field, the metallic material’s domains align, temporarily causing the metal to behave like a permanent magnet. This phenomenon is called magnetization. The intensity of this magnetization *M* can be calculated based on the magnetic susceptibility of the core material $\chi_{m}$ and the intensity of the external magnetic field *H* (12), produced by the coil. Another way to represent magnetization is through the core’s volume *V* and the magnetic dipole moment *m* (13).

$$M=\chi_{m}H$$ (12)

$$M=\frac{m}{V}$$ (13)

For practical purposes, the behavior of the core can be approximated to a coil. This way, it is possible to find the equivalence between the magnetic field generated by both elements. For example, (14) can represent the magnetic field at a point along the axial direction (*Z* axis) of a coil with N turns and of a permanent magnet, respectively, at a distance Z from the surface. Using this method, the effect generated by the magnetized core can be calculated using (1) to (11) by approximating it to a coil with N turns.

$$B_{z}=\frac{\mu_{0}NIA}{2\pi z^{3}}=\frac{\mu_{0}m}{2\pi z^{3}}$$ (14)

Another important characteristic of the core material is its magnetic saturation point. This corresponds to the maximum value of magnetic flux density that the core can generate, regardless of an increase in the external magnetic field (coil). In other words, as the product of current and number of turns in the coil increases, the magnetization of the core will rise until it reaches saturation. Beyond this value, an increase in current or number of turns in the coil will not affect the core. For the ASTM A36 steel, the saturation point corresponds to 2.16 T.

The total magnetic flux density produced by the electromagnet corresponds to the superposition of the contributions from the solenoid and the magnetized core.

#### Wire gauge of the winding.

To reduce the number of design variables, a maximum of one pound (lb) of wire was considered for the decision-making stage. Given the arrangement of 3 coils, each will use one-third of a pound of wire. Due to the power supply’s limits, each coil will be powered with a maximum of 10 A. As the wire gauge increases, its diameter decreases; thus, its weight per length unit decreases. This means that with a smaller gauge, a higher number of turns can be achieved with the same total weight of wire. However, increasing the gauge also increases the resistance of the wire, requiring a higher voltage to produce the same current intensity. Based on this, 20 AWG wires were selected. This gauge that has the smallest diameter still allows a 10 A current with less than 15 V (needing only 10.82 V), considering the wire length corresponds to one third of a pound.

#### Electromagnets dimensions.

**Fig 3:**
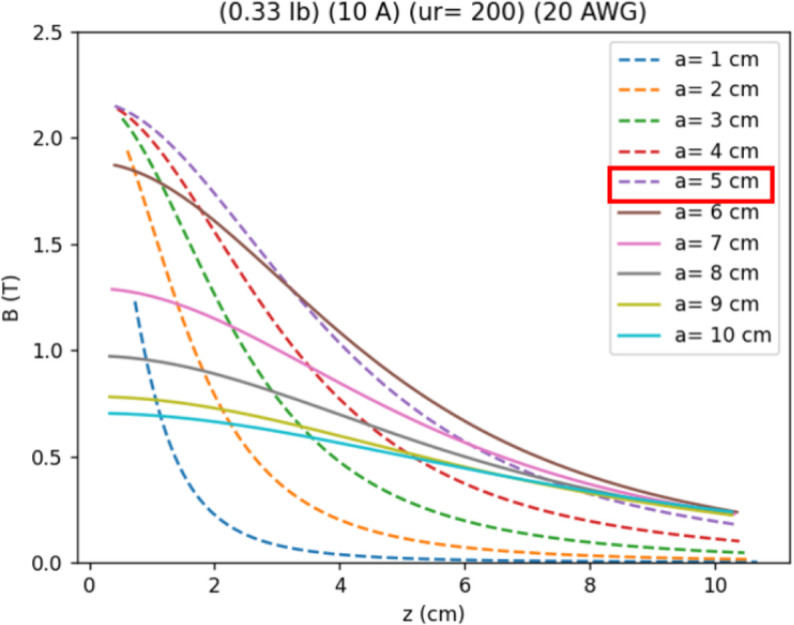
Magnetic flux density along the axis of a single electromagnet. Considering 0.33 pounds of 20AWG wire, supporting a current of 10A, and a low-carbon core with 200 units of magnetic permeability. The 5cm radio (red box) was chosen, considering it as the behavior with the higher area below the curve (segmented purple line).

A Python code was used to calculate the magnetic field along the Z-axis of electromagnets with different radii (*a*). For this, the previously defined or selected parameters were used. Fig 3 shows the curves describing the variation in the magnetic flux density at a point on the axis as it moves away from the surface of the electromagnet. The dashed lines represent the saturation state of the core. In other words, in these scenarios, the maximum magnetic flux density on the surface of the electromagnet has been reached. It can be observed that electromagnets with a radius of 5 cm exhibit the most favorable behavior. Smaller radii show an abrupt drop in the magnetic field as the distance increases. On the other hand, larger radii do not reach their saturation state, which means that the properties of the material are not being fully utilized. Additionally, this radius maintains an appropriate magnetic field magnitude even at the maximum distance of 10 cm from the surface of the electromagnet. Therefore, a radius of 5 cm was selected for each electromagnet.

The Python code is shared as S1 Code, including the mathematical model to design the electromagnets, and the calculations to get the result figures shown in later sections. The code allows the users to change the parameters according to their needs to get an array suitable for their context and requirements.

#### Manufacturing of the electromagnets.

**Fig 4:**
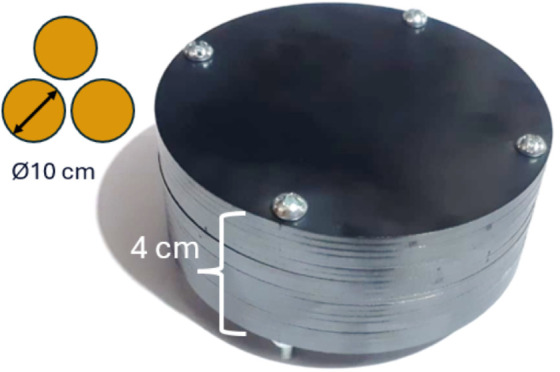
Ferromagnetic core dimensions. 5 cm radio and 4 cm height in a 3-electromagnet array.

The ASTM A36 steel is found mostly in the form of metal sheets. Therefore, the cores were constructed by stacking laser-cut discs from 2 mm-thick sheets of this material. Bolts and nuts fastened the discs. This way, a total height of 4 cm per core was obtained, as shown in Fig 4. This configuration was based on the laminated cores used in motors. It not only simplifies the manufacturing of the core but also increases its surface area. This improves heat dissipation, reduces hysteresis, and minimizes Foucault or eddy currents. As a result, the core’s efficiency increases, and energy losses are reduced.

Then, the 20 AWG wire was wound around the core. Aluminum tape was used to fasten the wire to the core. To reduce the heat generated by the high current in the coil, it was decided to triple the number of turns in each electromagnet. This allows using one-third of the considered current while maintaining the N*I product. In other words, the same behavior and magnitude of the generated magnetic field are kept. With this decision, each electromagnet was made with 300 turns, using one pound of wire per electromagnet. They were connected in parallel to the power supply to distribute a total of 10 A to the entire arrangement. Relay modules were used to command the activation of the electromagnet through a microcontroller signal. This way, the power and control circuits are electrically isolated.

**Fig 5:**
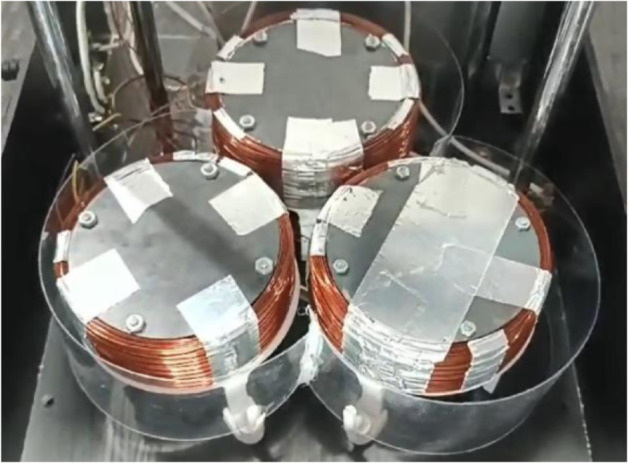
Electromagnets final array. Three electromagnets connected in parallel, with their ventilation system.

A simple ventilation system was designed to improve the lifespan of the electromagnets. The greatest heating occurred in the coil wire, while the core did not require ventilation due to its good heat dissipation. Hence, a 10 V fan was positioned below each electromagnet. Supports were designed so the electromagnets would not rest directly on the fans. The supports had a conical shape to redirect the airflow from the fan to the periphery of the electromagnet. This way, the wire is cooled rather than the core. A plastic profile was also used to surround the electromagnet array, concentrating the airflow in this zone and preventing it from dispersing outside the area of interest (Fig 5).

### Temperature control

Biochemical processes must be performed at a specific temperature to be fully efficient. Hydrolysis usually occurs between 45 °C and 55 °C. Lower temperatures would not allow the enzymes to work at their full potential, while higher temperatures would cause denaturation [20]. Similarly, each fermentation product has an ideal temperature range. For example, during red wine fermentation, the temperature must be kept between 24 °C and 29 °C. On the other hand, ethanol production should be carried out between 30 °C and 37 °C [21]. These ranges are acceptable for the process. However, maximum efficiency is achieved when the temperature presents the least possible variations. For the design of this subsystem, it was proposed to configure a PID controller capable of stabilizing a selected temperature between 25 °C and 50 °C, with an error of ±1 °C.

#### Temperature sensor.

**Fig 6:**
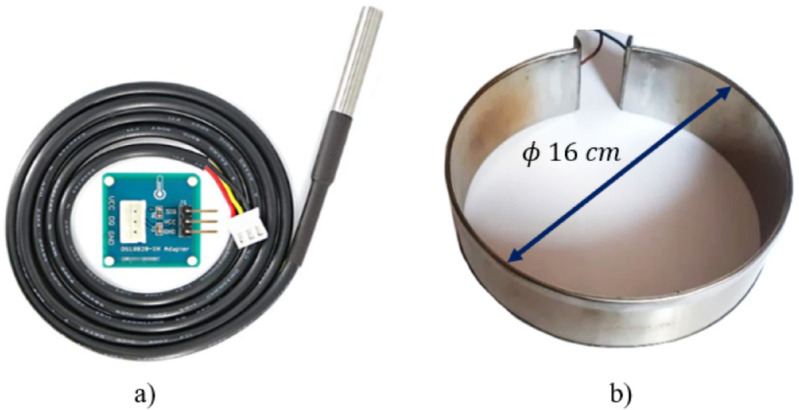
Temperature system components. A) DS18B20 temperature sensor. B) Armored resistor.

A temperature sensor was needed to create a closed-loop control system and recognize the current state of the substance. The DS18B20 sensor was chosen to get this feedback (Fig 6A). This digital sensor facilitates the acquisition and processing of the data. These kinds of sensors are less susceptible to electrical noise and induced currents. It includes a waterproof probe capable of being immersed inside the liquid to measure its temperature directly. It is easy to clean and sterilize, a desirable feature for the application studied. The sensor includes a module that simplifies the connection, requiring only three wires: power, ground, and data. It is also compatible with the Arduino programming language. The library *OneWire* was used for this project to get the sensor data.

#### Temperature actuator.

Different heating methods were evaluated. Since the array of electromagnets is located at the bottom of the container, it was not possible to place a heat source in that area. Therefore, a cylindrical armored resistor that wraps around the container (similar to a belt, as shown in Fig 6B) was chosen. To determine the dimensions of this resistor, it was necessary to calculate the maximum power *(P)* it should generate. For this, a mass *(m)* of 2500 g of water with an initial and final temperature of T_i_=2525 °C and T_f_=5025 °C, respectively, was considered. Assuming a heating time Δ of 5 minutes and the specific heat capacity of the water (*c*_*p*_), formula (15) was used to determine the resistor’s power capacity *(P)*. It also was assumed that heat transfer occurred exclusively by conduction. The resultant power was rounded to 1000 W, to consider possible energy losses. This data was provided to the manufacturer, resulting in a resistor with a height of 5 cm, a thickness of 5 mm, and a diameter of 16 cm to properly fit the container.

$$P=\frac{mc_{p}(T_{f}-T_{i})}{\Delta t}=870.97W$$ (15)

#### PID temperature controller.

Since the temperature is a very slow variable, the experimental tuning of the PID controller would have taken too long. Therefore, data was collected to approximate the behavior of the temperature subsystem.

**Fig 7:**
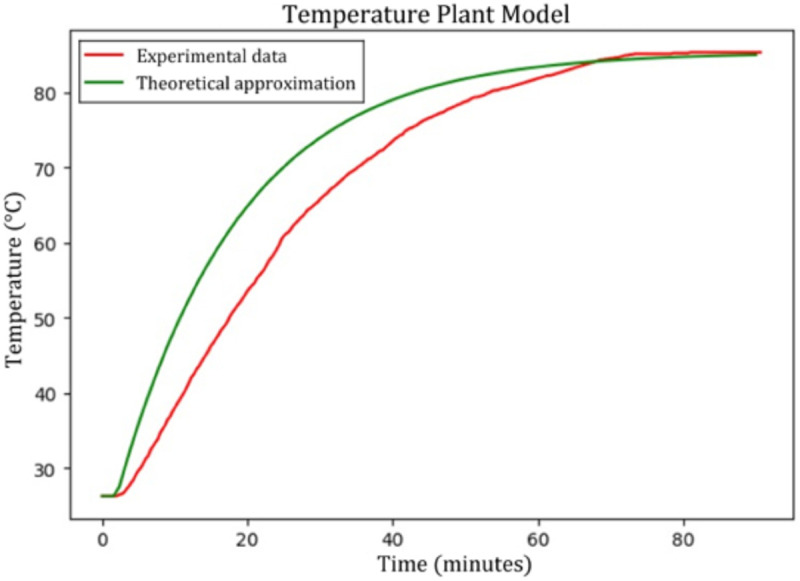
Temperature plant model approximation. Closest theoretical curve to the experimental data considering final temperature, stabilization time, and initial delay.

The sensor’s probe was submerged in the working substance while connected to a microcontroller to register the sensed temperature during the experiment. The armored resistor was connected to the power grid since it can work with AC current. Applying 120 V constantly to the resistor would generate a high stabilization temperature. This could be dangerous and harm the electronic components involved. Consequently, a solid-state AC relay was used to regulate the power supplied to the resistor. By programming a duty cycle of one-third in the relay (with 5 seconds), the resistor received an average voltage of 40 V. The data was collected from when the resistor was powered on until the liquid reached its stabilization temperature. Knowing this final temperature value, the stabilization time, and the delay at the input signal, it was possible to find the theoretical approximation of the open-loop temperature plant model (*G*_*T*_ in Eq 16 and Fig 7). A first-order system, with a small sigmoidal behavior at the beginning, represents this variable.

$$G_{T}(S)=\frac{1.475e^{-120S}}{990S+1}$$ (16)

A small block diagram was created in Simulink (Matlab) in order to simulate the approximated curve with the experimental data. Also, the Matlab program was used to tune the PID constants, simulating different iterations. Finally, the constants with the best response were determined as: P=3.3, I=0.0033, and D=82.5.

### pH control

Hydrolysis and fermentation must be performed at different pH levels according to the enzymes and microorganisms participating in the process. However, the biochemical processes themselves provoke variations in the substance’s acidity. Working outside the recommended range would diminish the process efficiency or harm the integrity of the immobilized systems. Therefore, it is important to control this parameter. Conventionally, pH regulation has been performed manually. The researcher submerges a pH meter to measure the liquid. According to the lecture, the investigator must add the appropriate substance to change the pH until reaching the desired value. This method is inefficient since it requires an investigator to supervise the process and do manual tasks. It usually has high operation times and is susceptible to human errors. Therefore, this project proposes a design to automate this process.

#### pH sensor.

**Fig 8:**
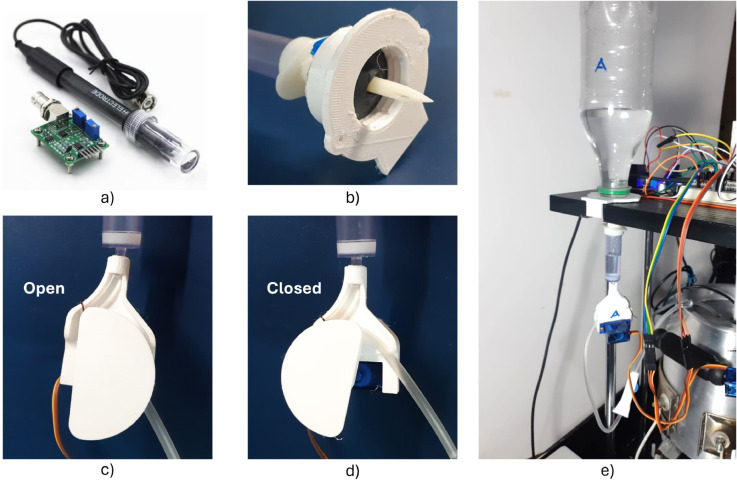
pH system components. A) pH0-14 BU0481 sensor. B) Sealed cap. C) Drip mechanism (open hose). D) Drip Mechanism (closed hose). E) Final pH control mechanism.

The pH0-14 BU0481 sensor was chosen to monitor the current acidity of the system (Fig 8A). It includes a digital module compatible with many microcontrollers like Arduino, being easy to program. It has a probe submerged in the liquid to do the measurement. Before using it, the sensor must be calibrated. For this, three standard samples were used: an acid pH4 solution, an alkaline pH10 solution, and a neutral pH7 solution.

#### pH regulation.

One of the most common techniques to regulate the pH is adding drops of a hydrochloric acid solution (HCl) or a sodium hydroxide alkaline solution (NaOH) to decrease or increase the pH of the working substance, respectively. Different solution concentrations were tested to observe the pH stabilization time and the sensor’s time response. This way, the concentrations were chosen, and HCl (0.47M) and NaOH (0.5M) solutions were prepared.

Two bottles of each chosen solution are placed above the main container. A drip mechanism was designed to dispense these regulatory substances. It consists of an adaptation of a serum drip chamber. Using a servo motor, the hose is strangled to regulate the flow. Fig 8C and 8D show the drip mechanism in its open and closed position, respectively. The whole pH mechanical system installed in the prototype is shown in Fig 8E. This mechanism was printed using PETG (polyethylene terephthalate glycol) due to its higher mechanical and temperature resistance than other plastics like PLA (polylactic acid).

Based on the previous tests, it was decided that the “open” configuration would correspond to a flow of one drop per second. Therefore, the system was programmed to sense the current pH value. The appropriate mechanism will be activated for three seconds if the pH exceeds the acceptable range. Then, the chamber would close again, waiting 5 seconds for the medium to mix correctly and the sensor to read the stabilized pH. After that, if the pH is still outside the desired range, the described process will be repeated.

Sealed caps were also manufactured to avoid bottle leaks and guarantee sterility. They were printed to be adaptable to different kinds of plastic bottles and be easy to install and disassemble from the prototype (Fig 8B). They used the rubber seal in the serum bottles to secure the connection between the bottle and the drip chamber.

### Agitation speed control

The hydrolysis or fermentation culture must be stirred to distribute the immobilized systems in the medium properly. Most processes require low agitation speeds, under 100 RPM. Therefore, the control system was designed to keep a constant velocity with a chosen value between 25 and 100 RPM.

Usually, food applications prefer magnetic stirrers. However, this would interfere with the magnetic field generated to attract the immobilized systems. Hence, a mechanical Rushton stirrer was selected. It is one of the most common and easiest to manufacture. The preferred material for this component is stainless steel. However, its manufacture was too expensive, and the time required exceeded the project’s planning. Instead, the stirrer was 3D printed with PETG. This material is easy to sterilize and works efficiently without deformation at the working temperatures considered (50 °C). It also had the appropriate mechanical resistance to avoid fracture or deformation due to the torque generated from the stirring motion itself.

**Fig 9:**
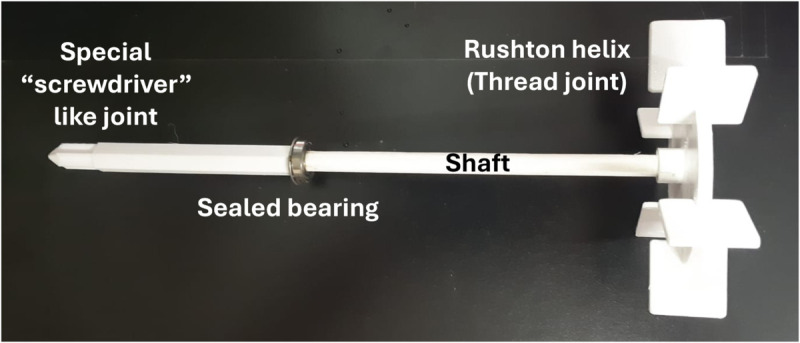
Stirrer design. 3-piece PETG detachable Rushton stirrer.

Two parts form the stirrer (shown in Fig 9). The helix, which consists of a disc surrounded by six squared paddles, is responsible for mixing the product. The shaft connects and transmits motion from the motor to the helix. A threaded joint connects the shaft and the helix. At the other edge, the shaft is connected to the motor by a joint shaped like a screwdriver. This allows the assembly and disassembly of the shaft and the motor by aligning both axes and performing a vertical movement. It doesn’t matter which angular position the motor stops; its joint will always fit. In addition, a sealed bearing was used to hold the shaft on the main container’s lid.

The right motor was chosen according to the desired power. First, the Reynolds number *(R*_*e*_) was calculated using (17), considering the water dynamic viscosity μanddensityρas an approximation to the working substance characteristics. Also, the stirrer diameter D_i_=0.1 m and the rated speed N_i_=100 rpm are involved. The resulting value was R_e_=16670 units, classifying the working substance as a turbulent liquid.

$$R_{e}=\frac{\rho N_{i}D_{i}^{2}}{\mu }$$ (17)

Hence (18) is applicable, considering the power number N_p_=5, according to standardized curves for this kind of stirrer. The calculated power was P=0.23W. It is a theoretical value that assumes that the power needed to move the stirrer itself is too small compared to the power to mix the liquid. Nevertheless, in this stage, the liquid volume is too small. Hence, the stirrer power cannot be discarded. The motor was oversized to consider this and compensate for losses and external perturbations. This way, a GA25-370 DC motor, with a 3.5 W power rate and a 100 RPM-rated load speed was selected. It includes an integrated encoder that gets the feedback to control the velocity. The LN298 H bridge also controlled the velocity through a PWM signal.

$$P=N_{p}\rho N_{i}^{3}D_{i}^{5}$$ (18)

To automatize the disassembly of the system for its cleanup, a small motor was connected to move the bigger motor vertically, using a simple pinion rack coupling. Its function was to separate the shaft from the main motor, allowing it to take away the container from the whole system. When the container is placed again (verifying that the axes are aligned), the mini-motor will help the main motor to go down and connect to the stirrer again. Limit switches were placed at both edges of the motion.

Once the agitation subsystem was assembled, a PID controller was designed to stabilize the required speed. An experimental tuning method was performed using the working substance and the whole device setting to find the controller parameters. The resulting PID parameters were: P=0.18, I = 0.8, and D=0.0016

### Multitasking system integration

**Fig 10:**
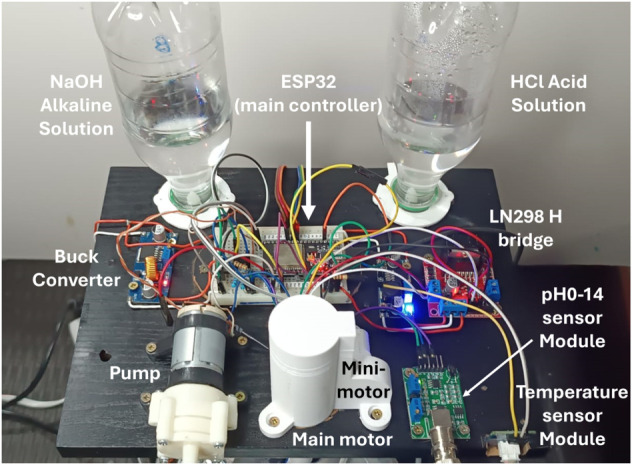
ESP32. Main microcontroller of the system. It is connected to all I/O devices, peripherals and auxiliary modules that allow interaction with the environment and management of the system.

To integrate all these subsystems, an ESP32-38pins was chosen as the main microcontroller of the system. It is a good choice due to its low cost and ease of acquisition. All the sensors and actuators previously selected are compatible with this technology. Also, most Arduino libraries are available for this board. Fig 10 shows how all components are connected to the ESP32 to integrate the multitasking system.

It was preferred over Arduino boards because of its higher clock frequency (80MHz, at least 5 times higher than Arduino UNO’s) and its dual-core processor. This was an important characteristic for optimal multitasking performance. Some variables, like the agitation speed, change too fast, meaning they need a higher feedback frequency to stabilize the value. On the other hand, slow variables like temperature and pH need more time to stabilize their current state lectures. While using an Arduino UNO, it was noticed that the system could not control all the variables simultaneously. The response time of the temperature and pH sensors was too high. By the moment the program returned to the speed control, the error was too big, causing multiple fluctuations. Therefore, it was decided to use an ESP32 and separate the subsystems between the two cores. The library FreeRTOS was used to create multiple threads (one for each task or variable) in both cores. The division was decided according to each subsystem’s need to distribute the available computational resources properly. This way, all the tasks were performed concurrently without delaying each other.

The code uploaded to the ESP32 to coordinate the multivariable system is provided in the file S2 Code.

**Fig 11:**
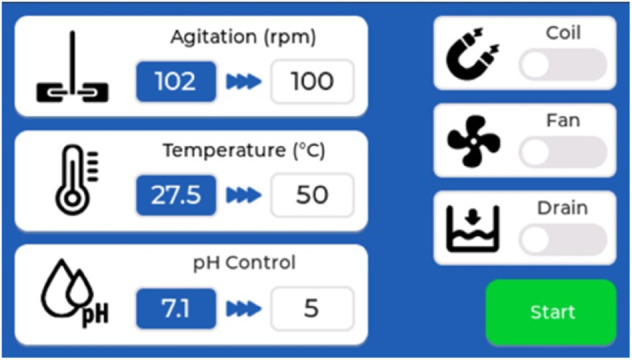
User interface main screen. It allows to see the current status of each variable and change their setpoints, access each variable dashboard, activate the main actions (magnetic field, draining, ventilation), and turn on/off the system.

The final user will be able to command all the tasks through a user interface. For this, the Elecrow Wizee-ESP32 WZ4827R043 4.3 in touchscreen was selected. It has an ESP32 integrated, which can be communicated to the main ESP32 through the serial UART port. The free trial of the Squareline Studio software was used to program the screen. This screen allowed the user to set the desired value of all controlled variables. The screen ESP32 sends the input data to the main ESP32 and receives the current values to display. It has secondary screens where a curve of each variable changing across time can be seen. This allows the investigator to see the current value, analyze the dynamics of the system, and save the history for further interpretation. The screen was configured to present the options to turn on the fans, the pump, the magnetic field generator, the assembly of the agitator, and start the whole control system. For safety, the fans could not be turned off when the electromagnets were on. Fig 11 shows the main screen of the user interface, from which the researcher can access each variable dashboard.

## Results and discussion

The laboratory equipment was fully assembled. It included all the electronic components like relays, drivers, and protection devices. The structure design considered the required ventilation for all the components, especially the power source. The device is connected to the power grid, and a switch enables the activation of the user interface to command the whole system.

All the subsystems were tested to validate the designed prototype. These experiments were all performed using the working substance (water + carboxymethyl cellulose). The orders were sent to the system through the user interface, where all the setpoints were defined. Temperature, pH, and agitation subsystems were tested together to evaluate the ability of the system to simultaneously control the involved variables, as it would be needed in a real hydrolysis or fermentation experiment. The following sections will describe the results of these tests for each variable.

### Magnetic field results

First, calculations were made to characterize the magnetic flux density generated by the manufactured electromagnets. The contributions of the three electromagnets were overlapped using Python code to find the resulting magnitude. These calculations were based on the equations shown in the Methods section. The distance between each wire layer was assumed to depend only on the wire gauge.

**Fig 12:**
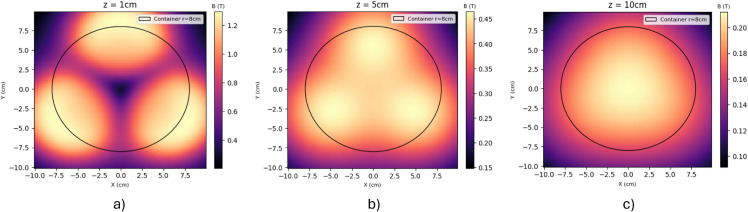
Magnetic flux density at a parallel plane. A) 1 cm height, B) 5 cm height, and C) 10 cm height.

Fig 12 shows the magnetic flux density magnitude distribution at a height of 1 cm, 5 cm, and 10 cm from the top of the electromagnets array. The plane *XY* is always parallel to the electromagnets’ surface, and *z* is the distance between that plane and the array. The color scale demonstrates where the magnetic flux is more intense. Even at a height of 10 cm, the resulting magnetic flux density is above the desired value of 0.2 T. As expected, magnetic flux density is higher at the bottom of the container due to the proximity to the electromagnets. Here, the magnetic flux density reached a maximum value above 1.2 T. However, the magnetic flux is less uniform at this height, showing darker spots that symbolize smaller magnitudes (according to the color scale). This happens because of the interaction of the different magnetic fields generated by each electromagnet. The darker spots are areas where the sum of all the different magnetic fields is too small. Also, zones where the different magnetic fields have opposing directions show smaller magnitudes due to the cancellation between these vectors. As seen, the magnitude of the magnetic flux density becomes more uniform and homogeneous as the height increases. This behavior is convenient since when the immobilized systems are farther from the base, it ensures that they all experience a similar magnetic field, being attracted equally. Once they are near the bottom, the magnetic field is strong enough to attract all the particles and direct them to the areas of higher magnetic flux density (brighter colors on the scale).

The expected behavior of the system is shown in the animation S3 Video. This shows the discharging of the main substance while the immobilized systems are retained by the magnetic field.

Some tests were performed to visually evaluate the magnetic field’s action and validate these results. Since magnetic nanoparticles are too expensive, a substitute component named *developer* was used. It is a powder made of iron fragments, commonly used in photocopiers. This allowed the simulation of the magnetic nanoparticles to be more economical. The magnetic powder was mixed with the working substance. Then, it was stirred to distribute the particles inside the liquid properly. The tests were performed in a different glass container to allow us to take pictures of the particles under the magnetic field action. For these experiments, temperature control was not necessary. Hence, it was possible to use a conventional household glass dispenser for cold beverages.

**Fig 13:**
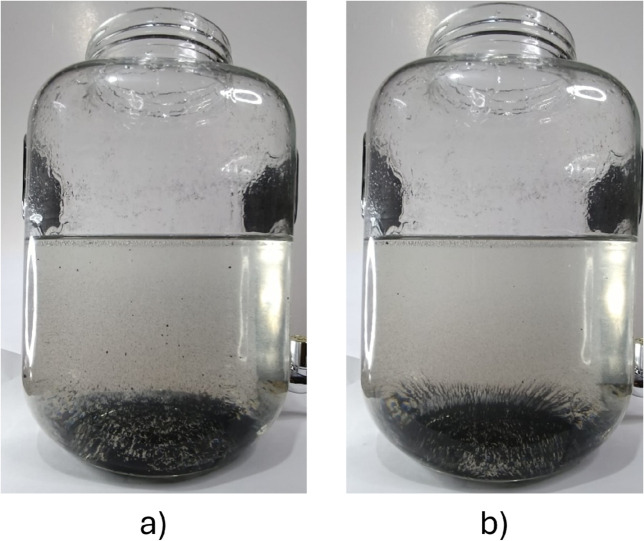
Decantation test. A) Without the magnetic field, B) Under magnetic field influence.

The first test consisted of mixing the working substance and the iron powder. The stirring stopped once the particles were properly distributed and the magnetic field system was activated. As a result, the particles started to descend to the bottom of the container. Images (Fig 13) were taken to compare the natural decantation provoked by gravity only, versus the decantation under the magnetic field action. Fig 13B clearly shows how almost all iron particles had been dragged to the bottom and aligned with the magnetic field. On the other hand, Fig 13A presents more iron particles that still float in the liquid. In the tests performed, it was noticed that the magnetic particles descended faster when the magnetic field was present.

**Fig 14:**
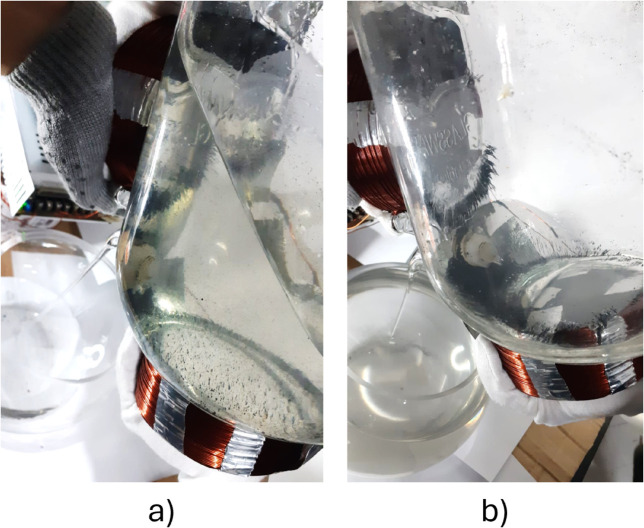
Draining test. A) Liquid being discharged, B) Discharge completed.

To reuse the immobilized systems, the equipment must be able to drain the liquid without dragging the magnetic particles outside the container. Fig 14 shows that the particles remained inside the container attached to the electromagnets when the liquid was drained through the dispenser’s tap. The same positive outcome was obtained when using the pump. The liquid was extracted while the magnetic particles were retained. This would not have been possible without the presence of the appropriate magnetic flux density. Therefore, the effectiveness of the magnetic field generator system was demonstrated. The electromagnets designed had the right capacity to retain the particles and enable their reuse. Once the magnetic field is turned off and the stirring begins, the immobilized systems spread through the substance, being able to participate in the new batch of fermentation or hydrolysis.

### Temperature results

The system was tested with different temperatures between 25 °C and 50 °C. It was desired to keep the overshoot and steady-state error below 1 °C. Higher deviations from the appropriate temperature could harm the enzymes or microorganisms. In temperature control systems, this acceptable range is considered very tight, especially considering the long times involved in the sensor response and the relay’s reaction. Nevertheless, the proposed controller got promising results.

The heat input is regulated varying the duty cycle (average voltage) of the armored resistor (actuator), as described in the methodology. There are three modes in the controller. First, when the current temperature is still too far from the setpoint, a maximum constant voltage is calculated and applied to the resistor. The PID controller is activated once the temperature has risen 20% of the desired change. Finally, the resistor is turned off when the desired temperature is reached. When the temperature descends from the setpoint, the PID controller is turned on again.

**Fig 15:**
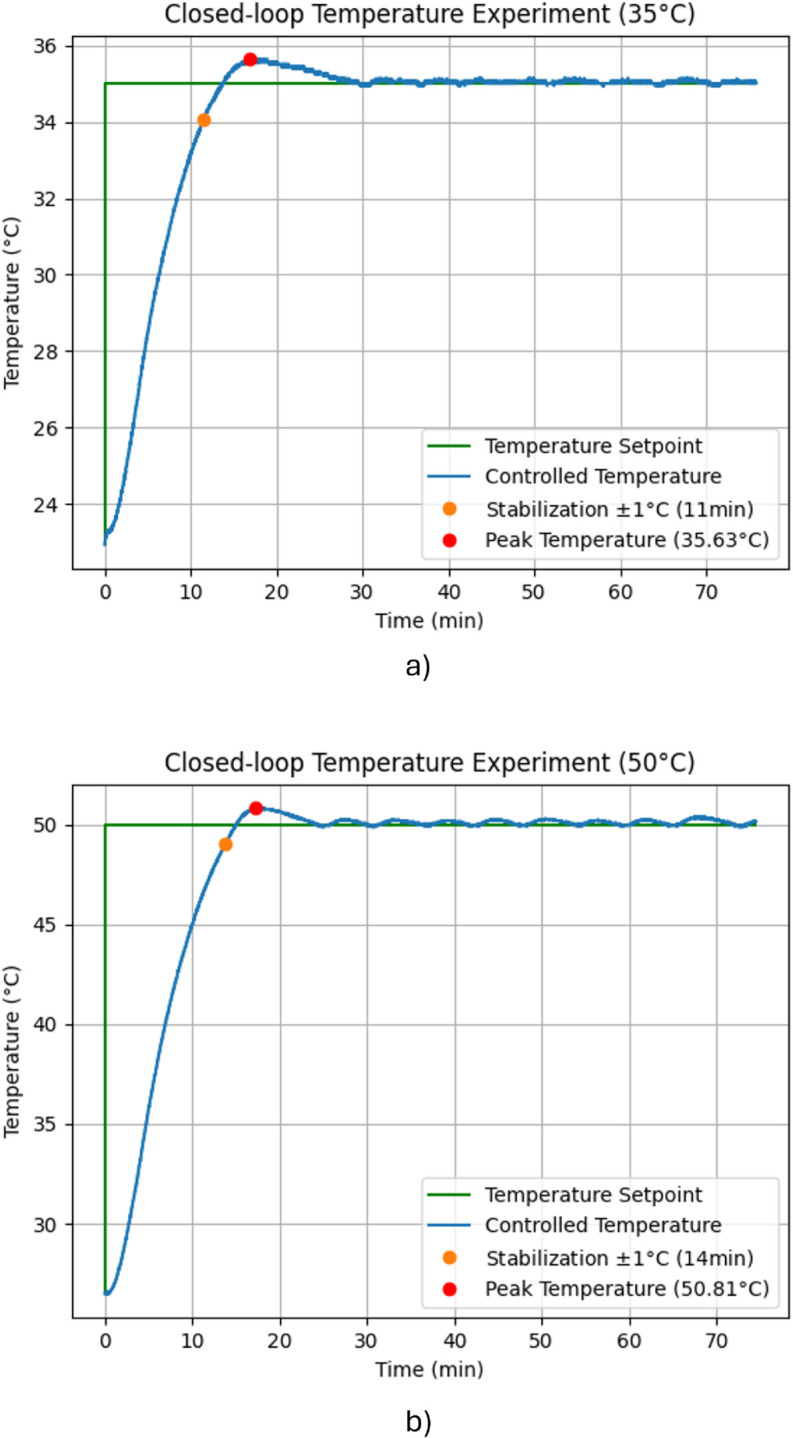
Temperature control results. A) Setpoint 35 °C, and B) Setpoint 50 °C.

Following this logic, the results for setpoints of 35 °C and 50 °C are shown in Fig 15A and Fig 15B, respectively. These values were chosen because they are very common in the biochemical processes. Each experiment was performed 5 times, showing in the graphics the corresponding average behavior. By comparing both curves, it is shown that the stabilization time, steady-state error, and overshoot are higher for the setpoint 50 °C. This was expected since even when the resistor is turned off, its residual heat keeps increasing the liquid’s temperature. This thermal inertia is higher when bigger changes in temperature are performed. The system can keep a temperature with minimal steady-state error in both scenarios. The overshoot was kept within the specified acceptable range. The stabilization time was defined for this context as the time when the absolute error remains below 1 °C. Therefore, average stabilization times of 11 min and 14 min were obtained, with a standard deviation below 1.5 min between the different experiments. This was considered a good response, since most fermentation or hydrolysis processes last between 24 to 48 hours. Hence, stabilization times represent a small percentage of the total process time. This demonstrated the effectiveness of the temperature control system that was designed.

### pH results

**Fig 16:**
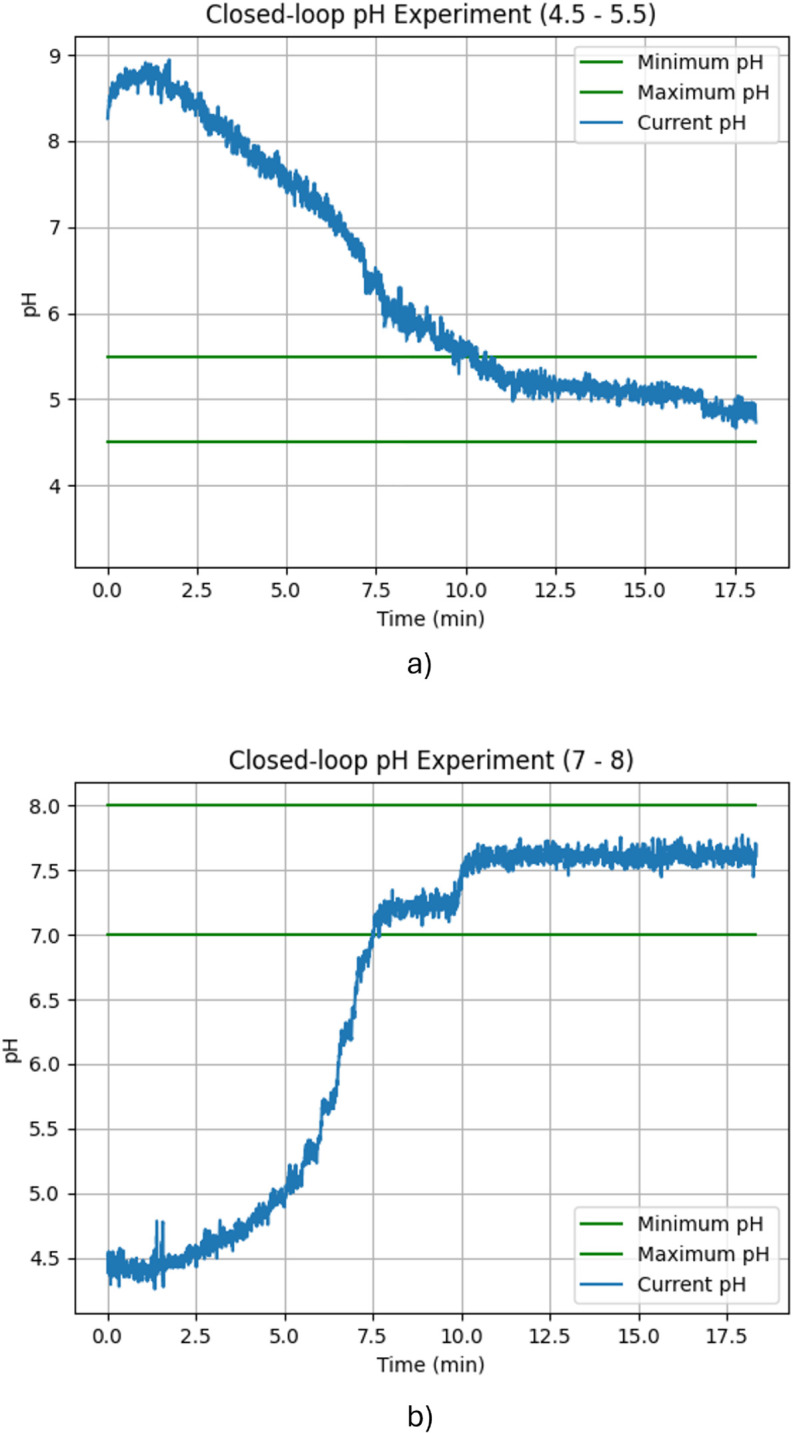
pH control results. A) Desired range 4.5 pH to 5.5 pH, and B) Desired range 7 pH to 8 pH.

The pH subsystem was tested considering a desired alkaline scenario with a pH from 7 to 8 units and a desired acid scenario with a 4.5 to 5.5 pH. The control was performed as described in the methodology. During the experiments, it was observed that the sensor was too sensitive to noise and vibrations from the stirrer. To compensate for this, a moving average filter was applied. This filter states a window size, calculates the average values inside the window, and considers this result to represent the corresponding period. Then, the window “slides” through time to filter the new upcoming data. Fig 16A and 16B were obtained by applying this technique 5 times for each scenario (acid and alkaline setpoint, respectively). It can be seen that there is still noise in the signal. However, the control system performs correctly. In both cases, the pH was stabilized to approximately the middle of the desired range. The average stabilization time was 10 minutes for the acid setpoint and 7.5 minutes for the alkaline setpoint. The standard deviation of each case was 1.2 minutes and 0.8 minutes respectively. Once again, these stabilization times are considered favorable since they represent a very small fraction at the beginning of the whole biochemical process This demonstrates that the multi-thread system allowed the proper control of many variables simultaneously, achieving the conditions expected.

### Agitation results

The motor’s angular velocity was the most susceptible variable for a multitasking system. The velocity is a very fast variable compared to the temperature and pH. The encoder used to calculate the current velocity needs to communicate fast and without interruptions with the main ESP32 to obtain accurate results. This was a difficult task when all subsystems were working in the same thread. It was noticed that the response time of the temperature sensor, pH sensor, and AC relay, were too long for the encoder. Therefore, we separated tasks across different threads and cores, depending on the computing resources and processing speed each task required. By using FreeRTOS, the issues mentioned before were solved. The encoder was able to continuously monitor the velocity. A moving average filter was also applied to minimize noise effects.

**Fig 17:**
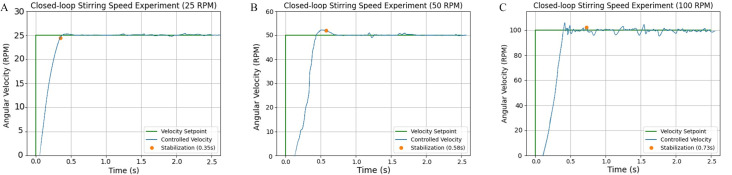
Stirring speed control results. A) Setpoint 25 RPM, B) Setpoint 50 RPM, and C) Setpoint 100 RPM.

For the applications exposed in this paper, stirring velocity needs to be near a reference value, but it is not necessary to keep it perfectly constant. Therefore, a steady state error of ±10 RPM was accepted. The curves of Fig 17A, 17B, and 17C, show the average performance of the agitation control after 8 tests with each reference velocities of 25 RPM, 50 RPM, and 100 RPM, respectively. Each experiment demonstrated a similar behavior. The system was able to adapt to each setpoint and reach the desired performance in a small stabilization time and low overshoot. The average stabilization time for each scenario is shown in the graphics, and the standard deviation for these values was around 0.2 seconds. It is also noted that the steady state oscillations increased at higher velocities. This is mostly because at faster motion the system generates more vibrations, inducing noise to the signals. Nevertheless, the controller was able to keep the velocity inside a tolerated range.

### Final prototype

The final product of this project was a complete open-source prototype for a bioreactor to perform tests of hydrolysis and fermentation in a controlled, automated, and documented way. As demonstrated above, all subsystems exhibited stable and favorable performance when operating simultaneously, without interfering with one another. This means that the proposed equipment is useful for studying fermentation and hydrolysis processes.

A complete view of the device with all its elements and subsystems working is shown in the S4 Video.

This was possible by using the FreeRTOS library to create a code with multiple threads. However, the use of this library is not enough to guarantee the effectiveness of a multi-variable system. For example, when FreeRTOS was deployed on Arduino UNO, the system still showed some failures, even when each task had its own thread. For this project to succeed, it was necessary to use a board like ESP32 with two cores available and a higher clock speed. The processing was developed faster on this board, compensating for the delays experienced due to sensor’s response times and actuators’ inertia. Also, being able to separate tasks between two cores allowed for a better distribution of computing resources.

The user interface allowed the user to insert the new setpoints, start or stop the subsystems, and monitor the state of the controlled variables. The secondary screens for each variable showed the corresponding curve in real time to allow the researcher to monitor the whole process and save a history for further analysis.

One of the most important design requirements was the ease of assembly and disassembly. The proposed prototype allows the user to perform these tasks comfortably and efficiently. All elements in contact with the working substance can be easily removed (sensor probes, hoses, stirrers, etc.). As explained, the agitator can be automatically decoupled by the action of the mini-motor that vertically displaces the main motor. After that, the main container can be easily removed. We attach the animation S5 Video with further details and steps on how to disassemble the BioMag 1 safely and properly.

As shown in the Fig 1, most of the electronic components are located on an upper platform, which does not obstruct the handling of the container. Besides the electromagnets, the lower area presented almost no devices in order to prevent them from being affected by magnetism or heat generated.

As mentioned throughout the article, this device was designed primarily using low-cost open-source technology. The main objective of our research was to provide researchers with a replicable tool that they can develop in their laboratories to support and enhance their own investigations. Basing the mathematical model on Python, one of the most popular programming languages, facilitates its review and understanding. The compatibility of the ESP32 with the Arduino system allows for the adaptation of affordable and easily accessible sensors and actuators available on the market, ensuring that almost any laboratory can source these components locally. This simplifies both manufacturing and maintenance, as well as the acquisition of spare parts for the equipment. Additionally, the handling, calibration, and connection of these devices are straightforward. The system’s programming is also simple, as it relies primarily on libraries provided by Arduino. Technical drawings for the replication of the designed mechanical components can be found in the Supporting information file: S6 Drawing.

**Repository:** https://github.com/DanielaS99/Biomag.git

During the development of this project, some limitations were encountered. As mentioned, this prototype was built with a modest budget, so certain components could be replaced to achieve better results. For example, the use of a stainless steel tank—although requiring a higher initial investment—could have a longer lifespan compared to the aluminum alternative used. Most electronic components, such as ESP32 boards and their compatible sensors, are very affordable and have proven to be highly efficient for this type of low-cost application. However, they are very delicate, and special care must be taken to prevent contact with any liquids, avoid cable movement, and ensure periodic calibration. Another element that may require adjustments is the mechanism used to administer acid or base for pH regulation. Since this regulation is mechanical, constant movement may cause the hose to shift, requiring periodic readjustments. A possible solution to this could be adopting a more sophisticated design, such as peristaltic pumps.

### Related work

Bioreactors that incorporate a magnetic field control to retain and reuse immobilized systems are still an emerging technology. Therefore, there is not a wide variety of them. As far as we could review, the studies conducted with this technique have been based on manual experiments, where the magnetic field is generated by permanent magnets, and agitation, pH, and temperature control are done manually. A notable study is that of [22], who developed a magnetic micro-bioreactor to retain immobilized systems. It is based on a permanent magnets array that holds the magnetic nanoparticles during the discharge of the liquid. That is, the separation process is performed outside the fermentation/hydrolysis chamber. This system is effective at attracting the immobilized systems and separating them from the final product. However, it does not automatize the reintegration of enzymes or microorganisms for reuse in the next batch. As explained earlier, our system addresses this deficiency.

Popular bioreactor designs were used as benchmarks to analyze the performance of other subsystems. It is worth emphasizing that our design presents an initial advantage by incorporating a magnetic field generation system, a feature absent in the following examples. Bioreactor models such as those described in [23–25] have demonstrated poor or imprecise temperature control, despite having similar dimensions to our design and targeting temperatures within the same range. The main cause of this is their intermittent mixing. In contrast, the system proposed in this paper maintains constant agitation, enabling rapid homogenization of the system’s temperature.

Other prominent studies on temperature control focus on the packed-bed family of bioreactors. These designs [26,27], primarily used in solid-state fermentation, employ a layered system. While they achieve favorable results, they require a more complex ventilation system to regulate the temperature and direction of incoming and outgoing air. Our system, equipped with elements specifically designed for the desired parameters, allows temperature control to be more easily achieved while maintaining precision.

Regarding pH regulation, most modern bioreactors only automate pH monitoring by displaying real-time readings, which require manual adjustments by an operator [28]. Few studies, such as that of [29], have focused on developing automated pH adjustment systems. The methodology followed in their study is similar to the one proposed in our design. They developed a controller capable of maintaining pH between 6.8 and 7.2 with a settling time of 2.6 seconds. However, that work remains theoretical and validated only through simulation. Considering the nonlinearity and disturbances present in a real-world implementation, we conclude that our results remain competitive and favorable in comparison.

Magnetic separation is an area of growing interest in this field. Many researchers have used this technique for the separation of microorganisms of interest, allowing, for example, the counting of bacteria in a medium. It also enables the detection of the presence of toxins, pathogens, or other agents of interest [30–32]. These projects have focused more on microbiological results but do not delve into the automation of the process. In addition, they do not specify a control system for the other variables involved in the process. Finally, unlike our system, they do not mention the reuse of these particles when introducing a new medium. As can be seen, other research usually focuses on improving a single subsystem. In contrast, our work presents a multitasking system that has been tested to demonstrate efficient control of all system variables simultaneously.

Table 1 shows a comparison with other studies. A topic is considered to be covered by the paper if it includes a proper description of how it is being implemented in the proposed system. It is noticeable that our work stands out as the only one to cover all the subsystems in a simultaneous way, with a completely automated multitasking control system. It is important to highlight that bioreactors are sophisticated laboratory equipment typically sold at very high costs. Depending on the number of sensors and actuators included, these systems can cost around $20000. Even a low-cost alternative proposed in [33] has a manufacturing cost of $3650 (without including a magnetic field generator system). In this regard, our design offers a significant advantage, being much more affordable with a cost of only $1080, less than one-third of the most economical alternatives available today. This supports the previously stated hypothesis regarding the economic benefits of open-source technology. Since 2012, the scientific community has been encouraged to develop open hardware. This has been made possible thanks to the availability of rapid prototyping technologies such as 3D printing, as well as development boards like Arduino, ESP32, and Raspberry Pi, which also support a wide range of affordable and compatible sensors and actuators. Many of these devices are marketed under the “Do It Yourself” (DIY) label [34].

**Table 1:** Comparison of state-of-the-art bioreactor projects.

Paper	Magnetic field	Temperature	pH	Agitation	Particle reuse
[22]	Automated				
[23]		Yes		Yes	
[26]		Yes		Yes	
[27]		Yes			
[28]		Yes	Yes	Yes	
[30]		Yes		Yes	
[31]	Manual	Yes	Yes	Yes	
Ours	Automated	Yes	Yes	Yes	Yes

The development of open-source hardware enables the creation of novel laboratory equipment and the customization of existing instruments or devices. These technologies offer good replicability and scalability, allowing any research team to access and adapt them to different projects. Prototyping generally involves risks such as non-functional designs, damage during testing or experiments, etc. These risks are mitigated by the low cost of the technology. Another advantage is the ease of repair and maintenance, as replacement parts can be sourced locally. Additionally, there is no need to rely on the time, availability, and warranties of manufacturers, as is the case with proprietary hardware [35].

The use of this type of technology in the field of Biology has become popular in recent years. The literature includes various laboratory instruments and equipment, such as sensors, mixers, incubators, etc. The possibilities for the development of open-hardware are endless, allowing the creation of a wide range of equipment using low-cost materials or reusing second-hand or obsolete instruments. Projects of this nature that promote technological development can range from simple tools like 3D-printed pipettes to complex equipment such as open-source thermocyclers or transilluminators, resulting in savings of thousands of dollars [15].

Based on this trend, we provide our design to support biochemical and biotechnological development focused on the food industry and energy generation.

## Conclusion

This project successfully designed and build *BioMag 1*, a prototype for conducting hydrolysis and fermentation tests, reusing previously immobilized enzymes and microorganisms. An electromagnet array was designed that is capable of generating the magnetic flux density necessary to retain the magnetic nanoparticles during liquid discharge and release them for reuse in the next batch. This behavior was represented through a mathematical model and a Python code capable of estimating the magnetic flux generated when different parameters are evaluated in the array.

In addition, subsystems for temperature, pH and agitation speed were designed, allowing precise control of these variables. All results were favorable, meeting acceptable error ranges and stabilization times. Consequently, a complete multitasking system was achieved, enabling researchers to perform controlled trials in an automated way. Moreover, users can monitor and control the system through the user interface. It was designed to be intuitive while providing a data history for all controlled variables.

The equipment also meets all design requirements, being easy to disassemble for cleaning and sterilizing. All relevant information to replicate this work is detailed in this document and in the presented repository.

*BioMag 1* presents multiple opportunities for future work. First, additional sensors could be integrated to monitor more parameters and display them on the touchscreen. CO_2_ and O_2_ are examples of relevant read-only variables that could provide important information to researchers. Based on laboratory needs, new versions of the equipment could be built from this original design to accommodate different dimensions. Finally, an industrial version of this system could be developed once the profitability of this technique is fully demonstrated.

## Supporting information

S1 Code Magnetic field. Python including the mathematical model of the electromagnets. Dimensions, materials, and other parameters can be changed to visualize different behaviors. The number of electromagnets can also be varied to analyze the interaction of different array configurations.
(ZIP)

S2 Code ESP32. Multitasking system code based on FreeRTOS. This code includes the reading of all sensors, control of all actuators, and communication with the user interface.
(TXT)

S3 Video Discharging animation. Expected behavior of the electromagnet array retaining the magnetic nanoparticles.
(MP4)

S4 Video Final prototype. Complete view of BioMag 1 built and working.
(MP4)

S5 Video Disassemble. Steps to remove sensors and the main recipient for cleaning them.
(MP4)

S6 Drawing Technical drawings. These files include dimensions of the 3D printed parts, a list of parts, and the electronic schematic diagram for all the connections needed.
(ZIP)
