# Supplementary material for: BioMag 1: A magnetic approach for efficient enzyme and microorganism reuse in biochemical processes for energy and food industries
Source: PLoS One. 2025 Dec 22;20(12):e0338444. doi: 10.1371/journal.pone.0338444 (PMC12721546; doi:10.1371/journal.pone.0338444)
Supplement: S6 Drawing — These files include dimensions of the 3D printed parts, a list of parts, and the electronic schematic diagram for all the connections needed. (ZIP) [file pone.0338444.s006.zip › Planos/PartList.pdf]

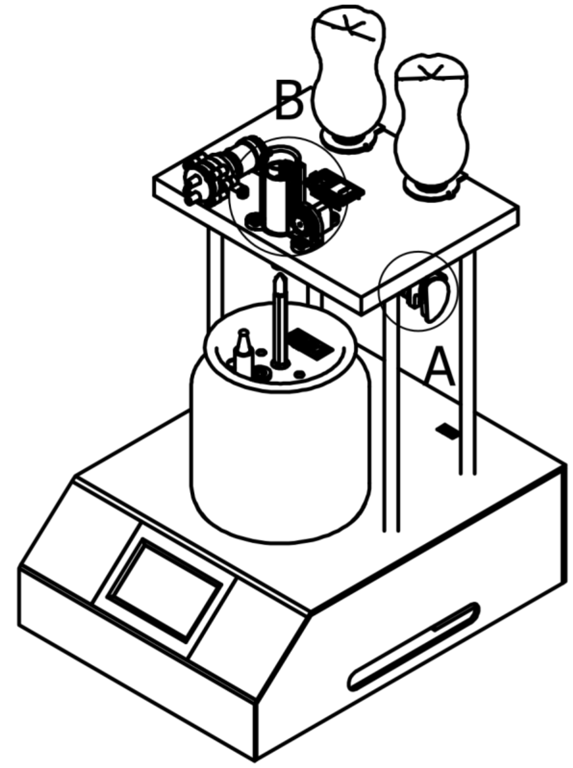

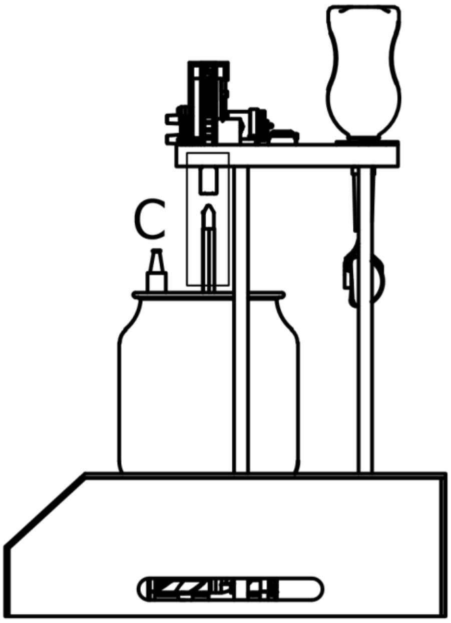

A (1 : 2)

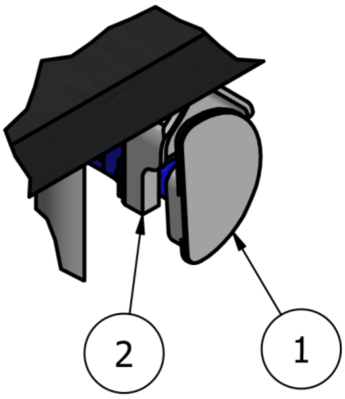

B (1 : 3)

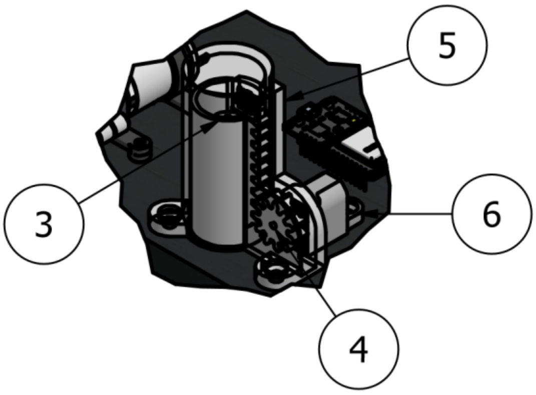

C (1 : 3)

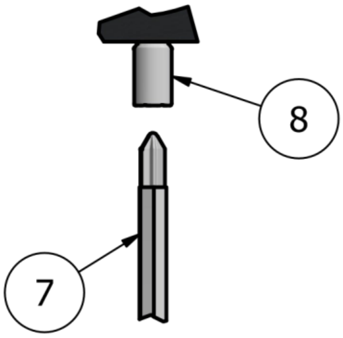

Part List		
**Part number**	**Quantity**	**Part Name**
1	2	Throttle Cam
2	2	Servomotor holder
3	1	Main motor Rack/Casing
4	1	Pinion
5	1	Safety guard (pinion-rack)
6	1	Safety guard (mini-motor)
7	1	Stirrer shaft
8	1	Stirrer shaft coupling

Daniela Sanchez-Orozco
Jerry Landivar
Socrates Palacios
Livingston Castro

Part
0

Biomag 1 (Part List)

Scale
1:7

Units
mm
