# Supplementary material for: BioMag 1: A magnetic approach for efficient enzyme and microorganism reuse in biochemical processes for energy and food industries
Source: PLoS One. 2025 Dec 22;20(12):e0338444. doi: 10.1371/journal.pone.0338444 (PMC12721546; doi:10.1371/journal.pone.0338444)
Supplement: S6 Drawing — These files include dimensions of the 3D printed parts, a list of parts, and the electronic schematic diagram for all the connections needed. (ZIP) [file pone.0338444.s006.zip › Planos/Pinion.pdf]

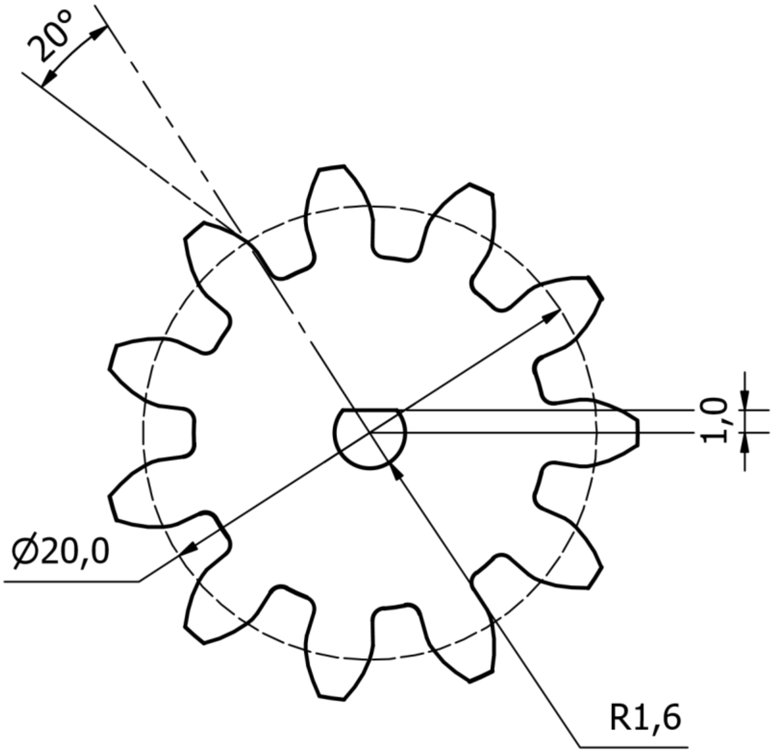

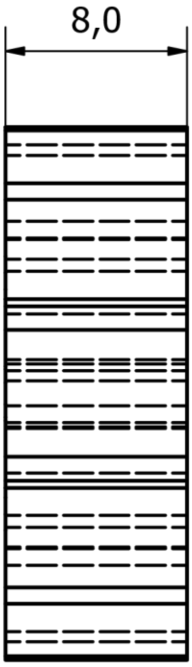

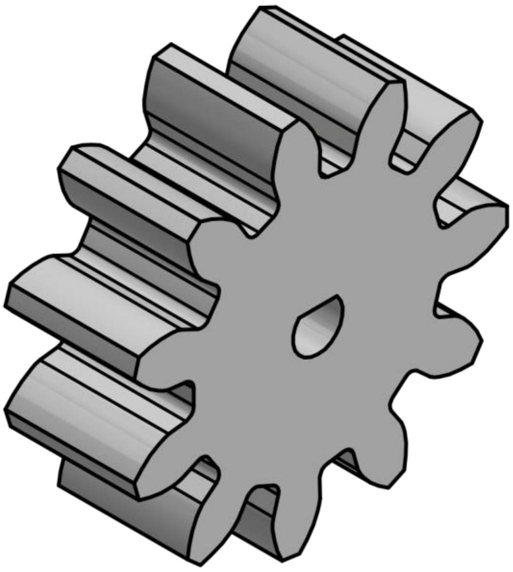

Daniela Sanchez-Orozco<br>Jerry Landivar<br>Socrates Palacios<br>Livingston Castro	Part<br>4	Pinion	
		Scale<br>1:7	Units<br>mm
